# Activation of 5-HT1A Receptors Normalizes the Overexpression of Presynaptic 5-HT1A Receptors and Alleviates Diabetic Neuropathic Pain

**DOI:** 10.3390/ijms241814334

**Published:** 2023-09-20

**Authors:** Neha Munawar, Milad S. Bitar, Willias Masocha

**Affiliations:** 1 Department of Pharmacology and Toxicology, College of Medicine, Kuwait University, Al-Jabriya 046302, Kuwait; neha_med@hotmail.com; 2 Department of Pharmacology and Therapeutics, College of Pharmacy, Kuwait University, Al-Jabriya 046302, Kuwait; willias.masocha@ku.edu.kw

**Keywords:** diabetic neuropathic pain, thermal hyperalgesia, cold allodynia, mechanical allodynia, 5-HT1A receptor, 8-OH-DPAT

## Abstract

Neuropathic pain is a well-documented phenomenon in experimental and clinical diabetes; however, current treatment is unsatisfactory. Serotoninergic-containing neurons are key components of the descending autoinhibitory pathway, and a decrease in their activity may contribute at least in part to diabetic neuropathic pain (DNP). A streptozotocin (STZ)-treated rat was used as a model for type 1 diabetes mellitus (T1DM). Pain transmission was evaluated using well-established nociceptive-based techniques, including the Hargreaves apparatus, cold plate and dynamic plantar aesthesiometer. Using qRT-PCR, Western blotting, immunohistochemistry, and HPLC-based techniques, we also measured in the central nervous system and peripheral nervous system of diabetic animals the expression and localization of 5-HT1A receptors (5-HT1AR), levels of key enzymes involved in the synthesis and degradation of tryptophan and 5-HT, including tryptophan hydroxylase-2 (Tph-2), tryptophan 2,3-dioxygenase (Tdo), indoleamine 2,3-dioxygenase 1 (Ido1) and Ido2. Moreover, spinal concentrations of 5-HT, 5-hydroxyindoleacetic acid (5-HIAA, a metabolite of 5-HT) and quinolinic acid (QA, a metabolite of tryptophan) were also quantified. Diabetic rats developed thermal hyperalgesia and cold/mechanical allodynia, and these behavioral abnormalities appear to be associated with the upregulation in the levels of expression of critical molecules related to the serotoninergic nervous system, including presynaptic 5-HT1AR and the enzymes Tph-2, Tdo, Ido1 and Ido2. Interestingly, the level of postsynaptic 5-HT1AR remains unaltered in STZ-induced T1DM. Chronic treatment of diabetic animals with 8-hydroxy-2-(dipropylamino)tetralin (8-OH-DPAT), a selective 5-HT1AR agonist, downregulated the upregulation of neuronal presynaptic 5-HT1AR, increased spinal release of 5-HT (↑ 5-HIAA/5-HT) and reduced the concentration of QA, decreased mRNA expression of *Tdo*, *Ido1* and *Ido2*, arrested neuronal degeneration and ameliorated pain-related behavior as exemplified by thermal hyperalgesia and cold/mechanical allodynia. These data show that 8-OH-DPAT alleviates DNP and other components of the serotoninergic system, including the ratio of 5-HIAA/5-HT and 5-HT1AR, and could be a useful therapeutic agent for managing DNP.

## 1. Introduction

Diabetic neuropathy is a highly prevalent condition affecting between 6 and 51% of patients with diabetes mellitus [1]. Painful diabetic neuropathy symptoms range from mild pins-and-needles sensations to burning, stabbing pain, or even unpleasant electric shock sensations and it is usually associated with hyperalgesia and/or allodynia [2]. Pharmacological treatment is the mainstay for managing painful diabetic neuropathy but is limited because of moderate efficacy and adverse effects of the drugs [3]. Therefore, understanding the complexity and likely multifactorial pathophysiological mechanisms behind painful diabetic neuropathy is thus needed for early diagnosis and appropriate personalized treatment.

The monoaminergic neurotransmitter serotonin (5-hydroxytryptamine; 5-HT) and its receptors are well known to be associated with descending pain modulation [4,5,6,7]. Serotonin exerts contrasting effects depending on the receptor subtype activated [8,9,10]. The 5-HT1A receptor (5-HT1AR) subtype, a G-protein-coupled receptor, is classically involved in modulating the transmission of nociceptive signals [11]. This receptor subtype is present on presynaptic and postsynaptic neurons and acts as both auto- and hetero-receptor. Furthermore, 5-HT1AR subtypes are expressed in the dorsal raphe nucleus [12,13], several brain regions [14] and on afferent nociceptive fibers in the dorsal horn of the spinal cord [1,15]. As an autoreceptor, 5-HTIAR also exists on the cellular soma and dendritic spines of the serotonergic circuit [16]. The main source for descending serotonergic inputs is the nucleus raphe magnus in the rostral ventromedial medulla (RVM). Activation of the RVM from higher brain centers leads to the release of 5-HT in the spinal cord dorsal horn (SCDH), which then acts on postsynaptic neurons and inhibits pain transmission [17,18].

Dietary tryptophan is metabolized via the methoxyindole and kynurenine (KYN) pathways; the earlier pathway metabolizes approximately 5% of the amino acid [19]. In the methoxyindole pathway, tryptophan hydroxylase (TPH) metabolizes tryptophan to 5-hydroxytryptophan (5-HTP). Then, 5-HTP is decarboxylated to 5-HT, which is metabolized by monoamine oxidase to 5-hydroxyindole acetic acid (5-HIAA). Therefore, synthesis of 5-HT depends on the availability of tryptophan and TPH activity [20]. Tryptophan hydroxylase comprises of two isoforms; isoform 1 is primarily expressed in the peripheral tissues, whilst the isoform 2 is present mainly in the neurons of the enteric and central nervous system, such as raphe nuclei of the brain stem [21,22]. In the KYN pathway, KYN is produced from tryptophan by the rate-limiting enzymes, indoleamine 2,3-dioxygenase (IDO) and tryptophan 2,3-dioxygenase (TDO) [19,23]. Kynurenine is metabolized to kynurenic acid, a primary neuroprotective metabolite [24,25] and quinolinic acid (QA), a key neurotoxic metabolite [23].

Although 5-HT1AR and α2-AR may be regarded as targets for therapeutic drugs, there is a lack of potential clinical candidates that have a high degree of selectivity towards either presynaptic and postsynaptic α2-AR or 5-HT1AR. Guanfacine, a selective α2A-AR agonist, and 8-hydroxy-2-(dipropylamino)tetralin (8-OH-DPAT), a selective 5-HT1AR agonist, have demonstrated therapeutic efficacy in models of pain [26,27]. Among 5-HT receptors, 5-HT1AR is characterized as antinociceptive [28]. Administration of 8-OH-DPAT, a selective 5-HT1AR agonist, decreased both nocifensive behaviors, including paw elevation and licking, in rats injected with formalin [26]. Furthermore, 8-OH-DPAT produced significant changes in sensorimotor functioning in a rat behavioral model for obsessive compulsive disorder (OCD). In the OCD study, 8-OH-DPAT elevated the response threshold to noxious thermal stimulus in the radiant heat test and decreased sensorimotor performance in the open-field test [29]. Another experimental study investigated the effect of antinociceptive and/or antidepressant drugs, including 8-OH-DPAT, duloxetine and fluoxetine, in a depression-like behavior model. In this study, treatment with 8-OH-DPAT improved depression-like behavior in rats with chronic constriction injury of the sciatic nerve. Both 8-OH-DPAT and duloxetine ameliorated mechanical hypersensitivity [30].

In view of these findings, we initiated the current study to examine the premise that diabetic neuropathic pain (DNP) stems at least in part from altered tryptophan/5-HT metabolism and that these diabetes-related abnormalities can be reversed by chronic administration of 8-OH-DPAT.

## 2. Results

### 2.1. Time– and Dose–Response Curves of the Antinociceptive Effects of 8-Hydroxy-2-(dipropylamino)tetralin (8-OH-DPAT) in Control and Type 1 Diabetes Mellitus (T1DM) Rats

Type 1 diabetes mellitus was induced with a single intraperitoneal (i.p.) injection of streptozotocin (STZ, 55 mg/kg). We have recently shown that diabetic rats developed hyperglycemia, exhibited decreased body weight, increased feed/water consumption and lowered nociceptive sensitivity in response to thermal (heat/cold) and mechanical stimuli when compared to those of control rats [27].

In order to study the acute effects and time/dose–response curves of 8-OH-DPAT, rats were treated with single separate doses of 8-OH-DPAT (0.25, 0.5 and 1 mg/kg, i.p.) at the same time. Nociceptive behavior in response to thermal (heat/cold) and mechanical stimuli were measured at different time points, for example, at 0, 15, 30, 60, 90 and 120 min after 8-OH-DPAT administration.

**Figure 1:**
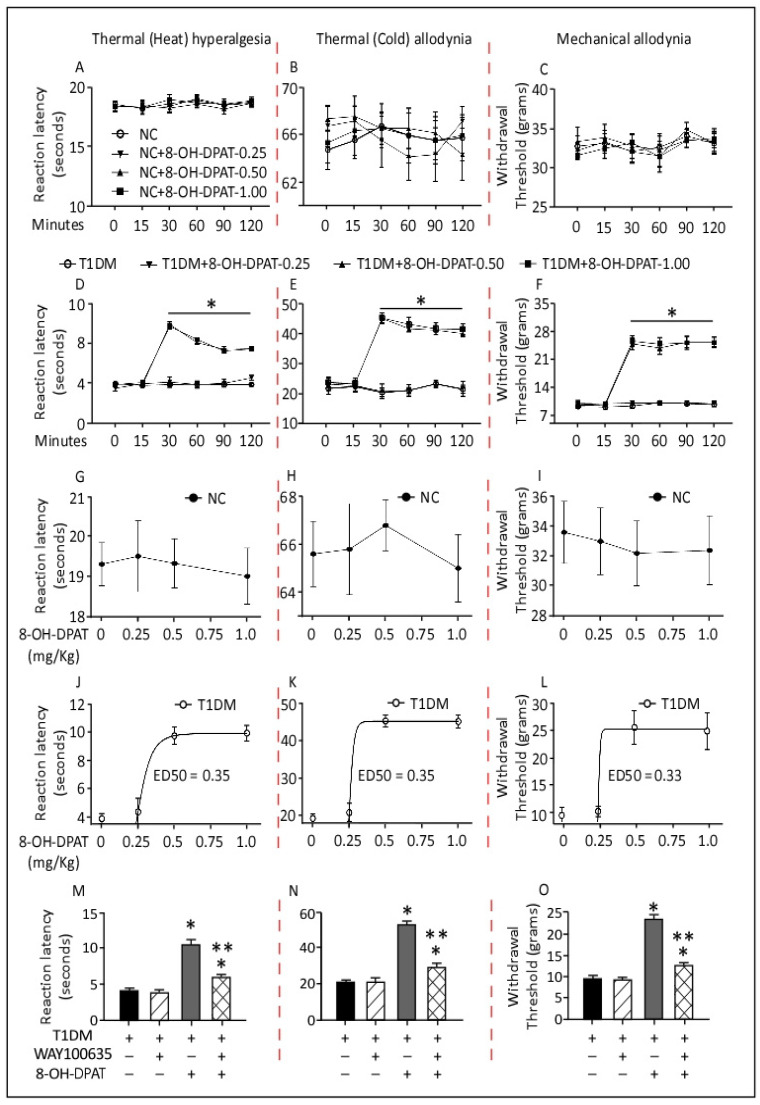
Time- and dose-response curves of administered 8-OH-DPAT in NC and T1DM rats. 8-hydroxy-2-(dipropylamino)tetralin showed no effect in naïve (**A**–**C**) rats but elevated nociceptive threshold dose-dependently in diabetic (**D**–**F**) rats. 8-hydroxy-2-(dipropylamino)tetralin elevated nociceptive threshold in diabetic in a dose-dependent manner (**J**–**L**) but not in control (**G**–**I**) rats at 30 min time-point post-administration. WAY100635 antagonizes 8-OH-DPAT’s antinociceptive effects (**M**–**O**). Each bar represents means ± SEM of values obtained from five animals/group. * 8-OH-DPAT-treated diabetic animals were significantly different from corresponding diabetic animals at *p* ≤ 0.05. ** Drug-treated diabetic animals were significantly different from corresponding 8-OH-DPAT-treated animals alone at *p* ≤ 0.05. Two-way ANOVA was followed by Bonferroni’s post-test, and one-way ANOVA followed by Dunnett’s post-test. Abbreviations: NC—normal control, T1DM—type 1 diabetes mellitus and 8-OH-DPAT—8-hydroxy-2-(dipropylamino)tetralin.

The administration of separate single i.p. doses of 8-OH-DPAT (0.25, 0.5 and 1 mg/kg) did not change the nociceptive threshold to the thermal (heat/cold) or mechanical stimuli (Figure 1A–C) in control rats. In contrast, treatment with 8-OH-DPAT induced antinociceptive responses to the tested stimuli in T1DM rats. One week following the STZ injection, 8-OH-DPAT was administered to T1DM rats. In these animals, administration of 8-OH-DPAT produced significant antinociceptive effects at 30 min, which persisted for up to 120 min following drug administration (Figure 1D–F, Hargreaves test: F: 35.1, *p* < 0.0001; cold-plate test: F: 12.5, *p* < 0.0001; dynamic plantar aesthesiometer: F: 24.9, *p* < 0.0001). The 0.25 mg/kg dose was ineffective, while 0.5 and 1 mg/kg doses demonstrated the same antinociceptive effect. Moreover, increasing concentrations of 8-OH-DPAT showed no changes in control rats to various aforementioned nociceptive stimuli 30 min after drug administration (Figure 1G–I). In contrast, antinociceptive responses were recorded in T1DM rats and the effective dose (ED50) for 8-OH-DPAT was 0.35 mg/kg in the Hargreaves and cold-plate test (Figure 1J,K) and 0.33 mg/kg in the mechanical (dynamic plantar aesthesiometer) test at 30 min following 8-OH-DPAT administration in T1DM rats (Figure 1L).

We further studied the effects of WAY100635, a 5-HT1AR antagonist, in T1DM rats. Treatment with only WAY100635 (1 mg/kg) did not change the reaction latencies and responses to withdrawal thresholds to thermal (heat/cold) and mechanical stimuli in comparison to baseline and control values (Figure 1M–O). However, WAY100635 (1 mg/kg) treatment was adequate to inhibit 8-OH-DPAT’s (0.5 mg/kg) anti-hyperalgesic and anti-allodynic effects. Treatment of T1DM rats with 8-OH-DPAT followed by WAY100635 significantly reduced (40–50%) the animals’ responses to the nociceptive stimuli in comparison to only 8-OH-DPAT, which, on the other hand, showed a significant increase in the rats’ responses to the various tested stimuli (Figure 1M–O, Hargreaves test: F: 12.3, *p* < 0.0001; cold-plate test: F: 10.5, *p* < 0.0001; dynamic plantar aesthesiometer: F: 13.8, *p* < 0.0001).

### 2.2. Persistent 8-OH-DPAT Antinociceptive Effects following Two Weeks of Withdrawal

**Figure 2:**
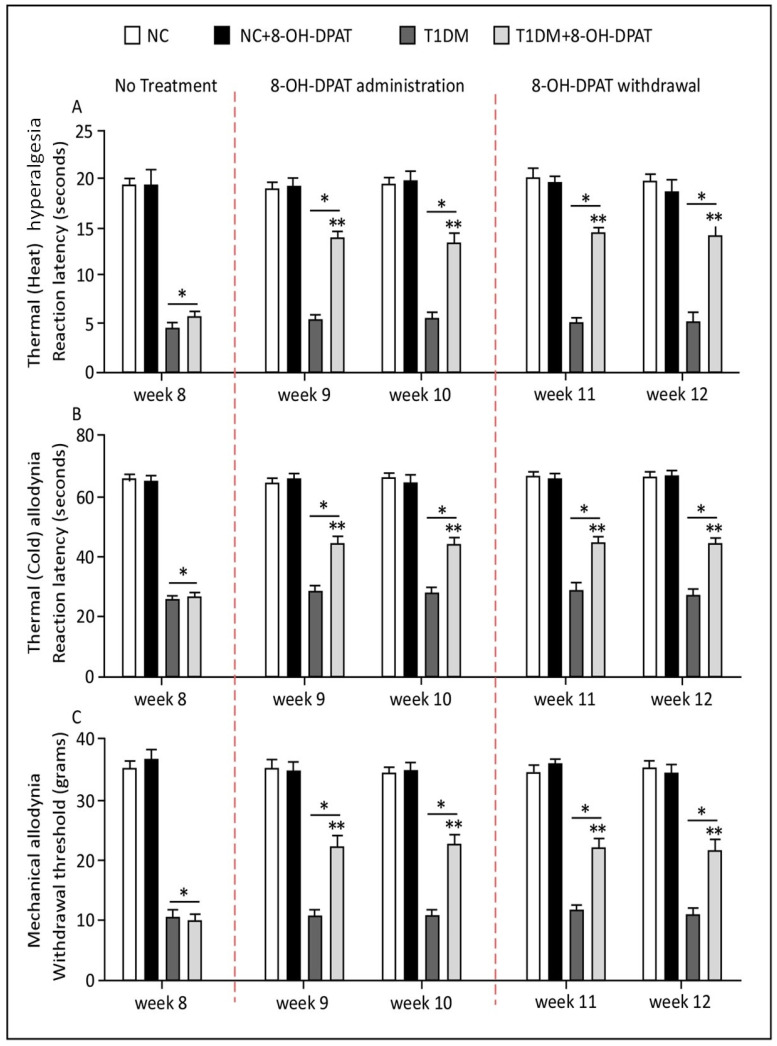
Persistent 8-OH-DPAT antinociceptive effects following two weeks of withdrawal. 8-hydroxy-2-(dipropylamino)tetralin (0.5 mg/kg) was injected i.p. on a daily basis for 14 days. Changes in nociceptive behavior to (**A**) thermal (heat), (**B**) thermal (cold), and (**C**) mechanical stimuli were assessed every week for 14 days in the presence and absence of 8-OH-DPAT. Each bar represents the means ± SEM of values obtained from five animals/group. * Diabetic animals were significantly different from corresponding control animals at *p* ≤ 0.05. ** 8-OH-DPAT-treated diabetic animals were significantly different from corresponding diabetic animals at *p* ≤ 0.05. Two-way ANOVA followed by Bonferroni’s post-test. Abbreviations: NC—normal control, T1DM—type 1 diabetes mellitus and 8-OH-DPAT—8-hydroxy-2-(dipropylamino)tetralin.

Following eight weeks of STZ injection, 8-OH-DPAT (0.5 mg/kg, i.p.) was administered chronically for a period of two weeks, and nociceptive behavior, as described previously, was assessed every week. Control animals did not show any change in responses to thermal or mechanical stimuli after chronic treatment with 8-OH-DPAT. On the other hand, chronic treatment of T1DM rats with 8-OH-DPAT elevated nociceptive (thermal heat) threshold by 2.4- and 2.7-fold in comparison to baseline and untreated values, respectively, following drug withdrawal (Figure 2A, Hargreaves test: F: 6.62, *p* < 0.0001). Likewise, treatment with 8-OH-DPAT elevated the nociceptive (thermal cold and responses to withdrawal thresholds) threshold specifically by 1.6- to 2-fold in T1DM rats, respectively (Figure 2B,C, cold-plate test: F: 5.43, *p* < 0.0001; dynamic plantar aesthesiometer: F: 4.64, *p* < 0.0001)).

### 2.3. Chronically Administered 8-OH-DPAT Arrests T1DM-Induced Neuronal Degeneration

**Figure 3:**
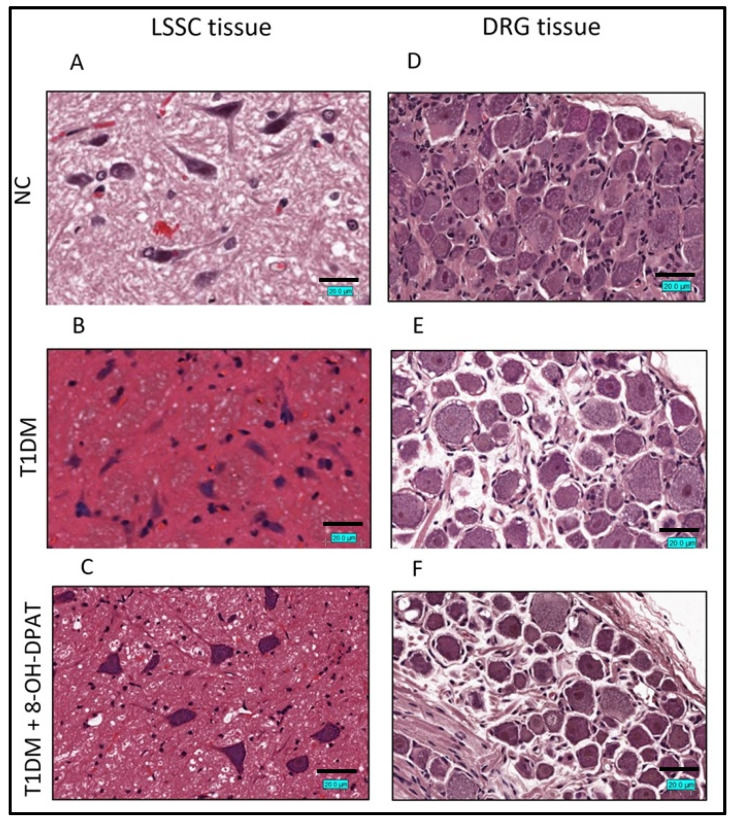
Chronically administered 8-OH-DPAT halts T1DM-induced neuronal degeneration. 8-hydroxy-2-(dipropylamino)tetralin (0.5 mg/kg) was administered i.p. on a daily basis for 14 days. Hematoxylin and Eosin staining of LSSC (**A**–**C**) and DRG (**D**–**F**), respectively (magnification: 40× and scale bar: 20 µm). Abbreviations: NC—normal control, T1DM—type 1 diabetes mellitus, LSSC—lumbar segment of the spinal cord, DRG—dorsal root ganglia and 8-OH-DPAT—8-hydroxy-2-(dipropylamino)tetralin.

Hematoxylin and Eosin (H&E) staining protocols were used to determine the effects of treatment with 8-OH-DPAT (0.5 mg/kg, i.p.) in STZ-induced T1DMin the lumbar segment of the spinal cord (LSSC, segment L1-L4) and dorsal root ganglia (DRG) of the spinal nerves tissue sections. Similar to what we observed before [27], the T1DM LSSC tissue sections revealed Nissl body loss and pyknosis, eosinophilic cytoplasm, ischemic neuronal cells, and glial cells with cellular edema (Figure 3B), which were not observed in those of control rats (Figure 3A). Furthermore, 8-OH-DPAT treatment improved the LSSC cellular structure but did not completely reverse it to the control LSSC histology (Figure 3C).

Similar to what we observed before [27], the H&E staining of the T1DM DRG tissue sections showed notable neuronophagia with basophilic, vacuolar-like defects and pigmentary inclusions, and satellitosis (Figure 3E) that was absent in the control DRG tissue section (Figure 3D). Moreover, treatment of T1DM rats with 8-OH-DPAT resulted in an improvement in the DRG tissue structure (Figure 3F).

### 2.4. Chronically Administered 8-OH-DPAT Reverses T1DM-Induced Upregulation of 5-HT1AR in the RVM, LSSC and DRG

**Figure 4:**
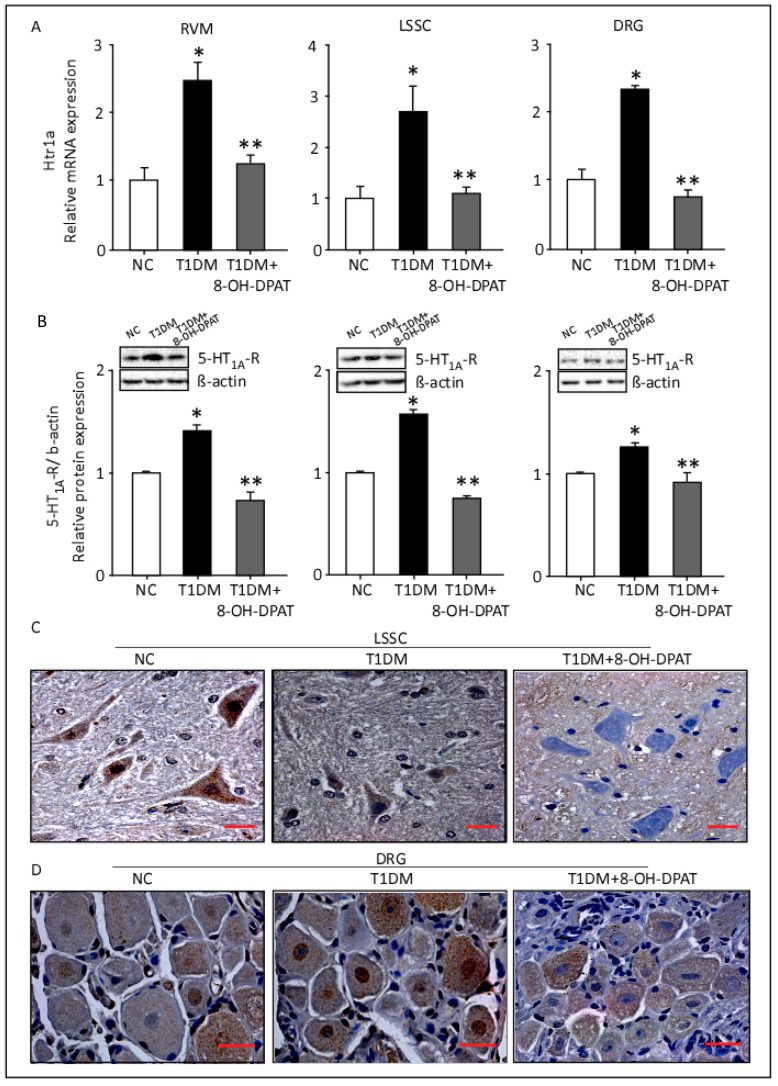
Chronically administered 8-OH-DPAT reverses T1DM-induced upregulation of 5-HT1AR in the RVM, LSSC and DRG. mRNA and protein expression of 5-HT1AR were assessed using qRT-PCR, Western blotting, and immunohistochemistry-based techniques, respectively. 8-hydroxy-2-(dipropylamino)tetralin (0.5 mg/Kg) was injected i.p. for 14 days. Relative mRNA expression of *Htr1a* (**A**). ImageLab 4.1 software was used to analyze protein bands of 5-HT1AR obtained with the ChemiDoc MP (**B**). Brown (immunohistochemical) staining pictures for 5-HT1AR expression in the LSSC (**C**) and DRG (**D**) tissue sections (magnification: 40× and scale bar: 10 µm). Each bar represents the mean ± SEM of values obtained from five animals/groups. * Diabetic animals were significantly different from corresponding control animals at *p* ≤ 0.05. ** 8-OH-DPAT-treated animals were significantly different from corresponding diabetic animals at *p* ≤ 0.05. One-way ANOVA was followed by Dunnett’s post-test. Abbreviations: NC—normal control, T1DM—type 1 diabetes mellitus, 8-OH-DPAT—8-hydroxy-2-(dipropylamino)tetralin, 5-HT1AR—5-HT1A receptors, RVM—rostral ventromedial medulla, LSSC—lumbar segment of the spinal cord and DRG—dorsal root ganglia.

A statistically significant increase in *Htr1a* transcripts in RVM, LSSC and DRG tissues was observed in T1DM rats when compared to control rats (Figure 4A). Remarkably, treatment with 8-OH-DPAT (0.5 mg/Kg, i.p.) normalized the levels of *Htr1a* mRNA in all the studied neuronal tissues of T1DM rats similar to those of control rats (Figure 4A, RVM: F: 13.3, *p* = 0.0062; LSSC: F: 6.96, *p* = 0.0273; DRG: F: 46.2, *p* = 0.0002). Similarly, Western blot results showed upregulation in the expression of 5-HT1AR in RVM, LSSC and DRG of T1DM rats in comparison to control rats (Figure 4B), which decreased significantly post 8-OH-DPAT treatment (Figure 4B, RVM: F: 25.7, *p* = 0.013; LSSC: F: 46.6, *p* = 0.0055; DRG: F: 7.8, *p* = 0.0048).

We further evaluated 5-HT1AR protein levels using an immunohistochemical staining technique. Immunoreactivity of 5-HT1AR proteins was more intense in LSSC cells of T1DM tissue sections than those of control tissue sections, whilst staining was decreased following treatment with 8-OH-DPAT (Figure 4C). A similar trend of 5-HT1AR protein immunostaining was also observed in the DRG tissue sections.

### 2.5. Expression of 5-HT1AR in the Presynaptic and Postsynaptic Fractions of the Spinal Cord Lumbar Region

**Figure 5:**
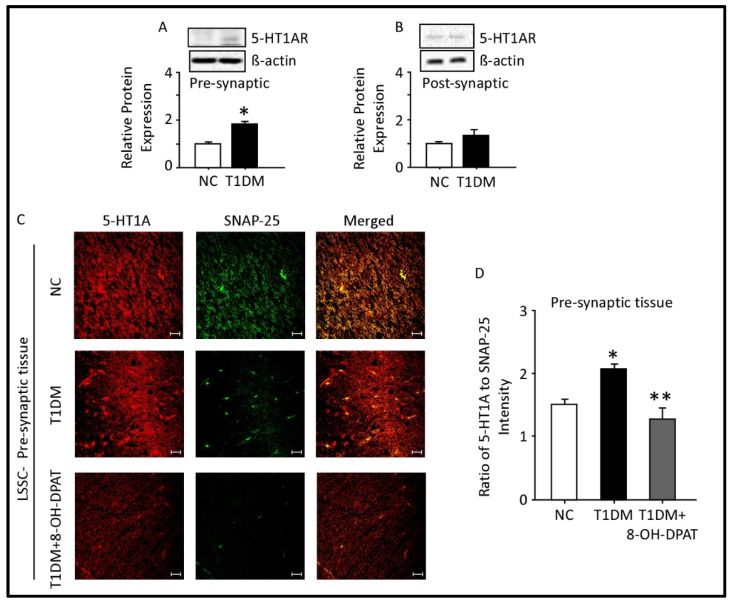
Expression of 5-HT1AR in the presynaptic and postsynaptic fractions of the spinal cord lumbar region. Type 1 diabetes mellitus upregulates presynaptic (**A**) but not postsynaptic (**B**) spinal 5-HT1AR proteins. Immunofluorescence localization of presynaptic (**C**) 5-HT1AR in control, diabetic and diabetic administered with 8-OH-DPAT for 14 days. Quantitation of fluorescence intensity of 5-HT1AR relative to SNAP-25 (**D**). ImageLab 4.1 software was used to analyze protein bands of 5-HT1AR obtained with the ChemiDoc MP. Data are expressed as the mean ± SEM of values obtained from five animals/group. * Diabetic animals were significantly different from corresponding control values at *p* ≤ 0.05. ** 8-OH-DPAT-treated diabetic animals were significantly different from corresponding diabetic animals at *p* ≤ 0.05. Student’s *t*-test: one-way ANOVA followed by Dunnett’s post-test. Representative confocal images at magnification: 40X and scale bar: 20 µm. Abbreviations: NC—normal control, T1DM—type 1 diabetes mellitus, LSSC—lumbar segment of the spinal cord, SNAP-25—synaptosomal-associated protein-25, PSD-95—postsynaptic density-95, 5-HT1AR—5-HT1A receptors and 8-OH-DPAT—8-hydroxy-2-(dipropylamino)tetralin.

Western blot analysis showed that the protein expression of 5-HT1AR was increased significantly in LSSC presynaptic fractions of T1DM rats (Figure 5A) but remained unchanged in the postsynaptic fractions (Figure 5B). T1DM lasted for a period of 12 weeks.

We recently showed that SNAP-25 is a biochemical marker for presynaptic LSSC tissues, while PSD-95 is for postsynaptic LSSC tissues [27]. Double-immunofluorescent staining with 5-HT1AR and SNAP-25 (Figure 5C) specific antibodies demonstrated expression and co-localization of these proteins in the LSSC presynaptic tissues. Quantitation of 5-HT1AR/SNAP-25 protein expression revealed a significant elevation STZ-induced T1DM (Figure 5C, *p* = 0.0491, *t* = 4.35). Chronic i.p. administration of 8-OH-DPAT (0.5 mg/kg) for a period of two weeks alleviated upregulation of presynaptic 5-HT1AR in LSSC (Figure 5D, F: 9.11, *p* = 0.0152) of STZ-induced T1DM.

### 2.6. Chronically Administered 8-OH-DPAT Reversed T1DM-Induced Dysregulation of Neuronal Spinal 5-HT1AR Signaling

**Figure 6:**
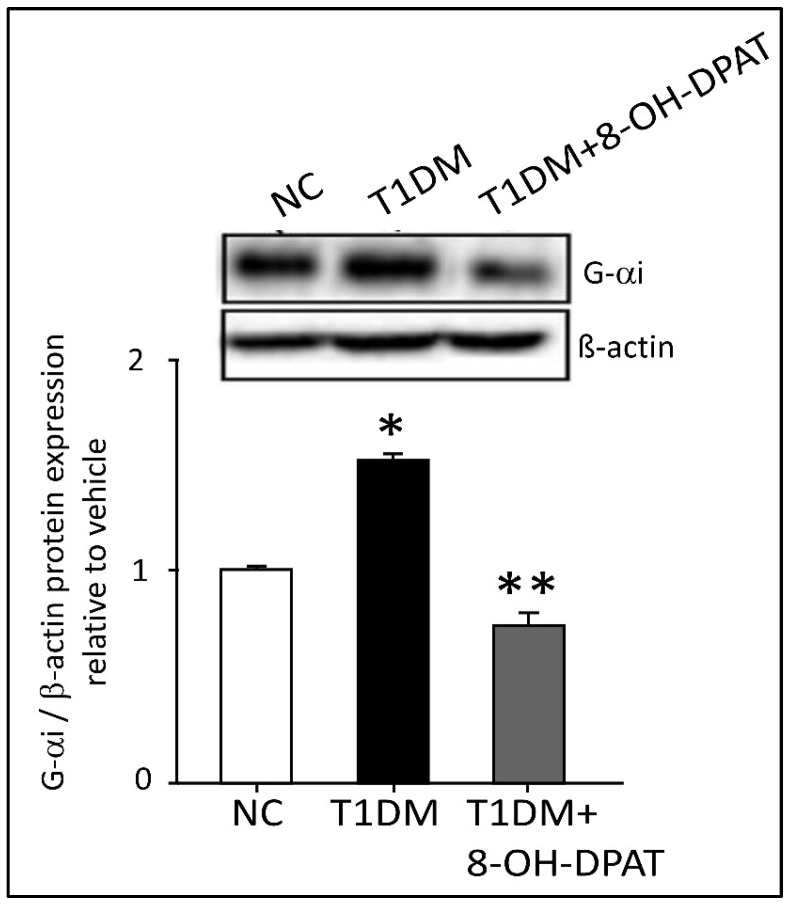
Chronically administered 8-OH-DPAT reversed T1DM-induced dysregulation of neuronal spinal 5-HT1AR signaling. G-α_i_ in the spinal cord lumbar region. 8-hydroxy-2-(dipropylamino)tetralin (0.5 mg/kg) was injected i.p. for 14 days. ImageLab 4.1 software was used to analyze protein bands of Gαi obtained with the ChemiDoc MP. Each bar represents the means ± SEM of values obtained from five animals/groups. * Diabetic animals were significantly different from corresponding control values at *p* ≤ 0.05. ** 8-OH-DPAT-treated diabetic animals were significantly different from corresponding diabetic animals at *p* ≤ 0.05. One-way ANOVA followed by Dunnett’s post-test. Abbreviations: NC—normal control, T1DM—type 1 diabetes mellitus, G-α_i_—G-protein coupled receptor G-alpha i and 8-OH-DPAT—8-hydroxy-2-(dipropylamino)tetralin.

5-HT1AR is a Gαi related receptor. In the LSSC tissue, the expression of Gα_i_ protein was significantly increased STZ-induced T1DM. Whereas treatment with 8-OH-DPAT (0.5 mg/kg, i.p.) significantly reduced the expression of Gα_i_ protein by 49.7% (Figure 6, F: 165.9, *p* = 0.0008).

### 2.7. Chronically Administered 8-OH-DPAT Normalizes the Upregulated Kynurenine Pathway, but Not the Methoxyindole Pathway

**Figure 7:**
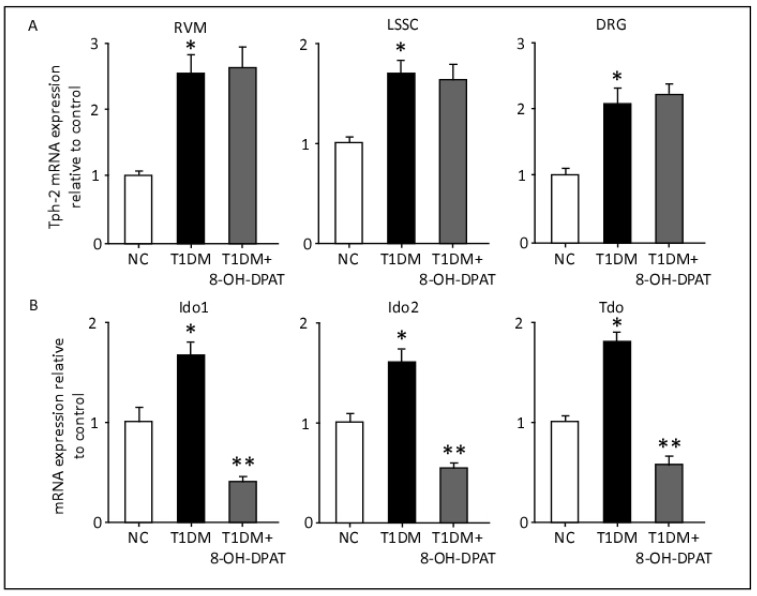
Chronically administered 8-OH-DPAT reverses T1DM-induced upregulation of *Ido1*, *Ido2* and *Tdo* but not *Tph-2*, in the spinal cord lumbar region. Expression of *Tph-2* (**A**), *Ido1*, *Ido2* and *Tdo* (**B**) were quantified at mRNA levels using qRT-PCR. 8-hydroxy-2-(dipropylamino)tetralin (0.5 mg/kg) was injected i.p. daily for 14 days. Each bar represents the mean ± SEM of values obtained from five animals/group. * Diabetic animals were significantly different from corresponding control values at *p* ≤ 0.05. ** 8-OH-DPAT-treated diabetic animals were significantly different from corresponding diabetic animals at *p* ≤ 0.05. One-way ANOVA followed by Dunnett’s post-test. Abbreviations: NC—normal control, T1DM—type 1 diabetes mellitus, RVM—rostral ventromedial medulla, LSSC—lumbar segment of the spinal cord, DRG—dorsal root ganglia, Tph-2—tryptophan hydroxylase-2, IDO—indoleamine 2,3-dioxygenase, TDO—tryptophan 2,3-dioxygenase and 8-OH-DPAT—8-hydroxy-2-(dipropylamino)tetralin.

In order to expand our knowledge of the role of the serotoninergic system in nociceptive behavioral responses, we evaluated the gene expression of the rate-limiting enzymes encompassing the methoxyindole and kynurenine pathways. As shown in Figure 7A, the gene transcripts of *Tph-2*, the rate-limiting enzyme in methoxyindole pathway, were significantly elevated in the RVM, LSSC and DRG tissues of T1DM rats in comparison to those of control (RVM: F: 11.02, *p* = 0.0098; LSSC: F: 7.27, *p* = 0.0249; DRG: F: 10.7, *p* = 0.0106). Treatment with 8-OH-DPAT (0.5 mg/kg, i.p.) did not alter the T1DM-induced elevation of *Tph-2* transcripts in the neuronal tissues (Figure 7A).

The gene expression levels of the kynurenine pathway enzymes *Ido1*, *Ido2*, and *Tdo* in the LSSC tissues were significantly upregulated in the T1DM rats in comparison to those of control rats (Figure 7B). Remarkably, treatment with 8-OH-DPAT significantly downregulated the expression of these enzymes in LSSC tissues, not only threefold relative to that in tissues from T1DM animals but also to levels below that observed in control animals (50–60% less, Figure 7B, *Ido1*: F: 32.6, *p* = 0.0006; *Ido2*: F: 19.6, *p* = 0.0023; *Tdo:* F: 29.9, *p* = 0.0008).

### 2.8. Effects of Chronic 8-OH-DPAT Administration on the Rate of Release of Serotonin (5-Hydroxytryptamine, 5-HT) as Reflected by the 5-Hydroxyindole Acetic Acid (5-HIAA)/5-HT Ratio and on the Levels of Quinolinic Acid (QA) in the Spinal Cord Lumbar Region STZ-Induced T1DM

**Figure 8:**
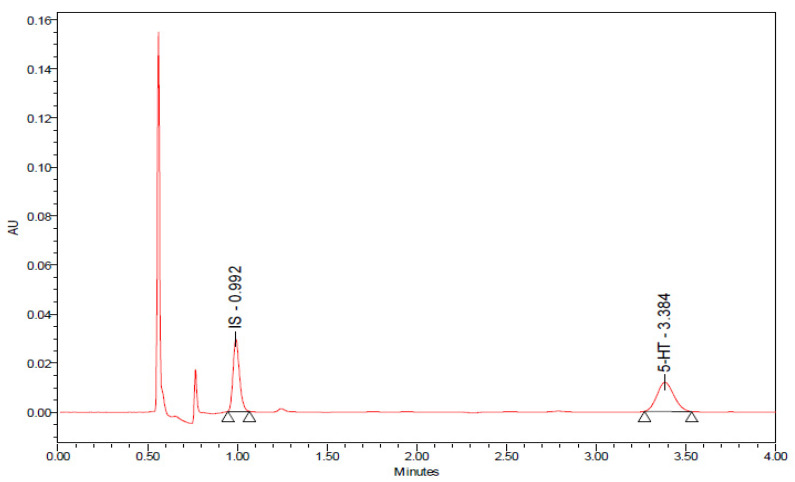

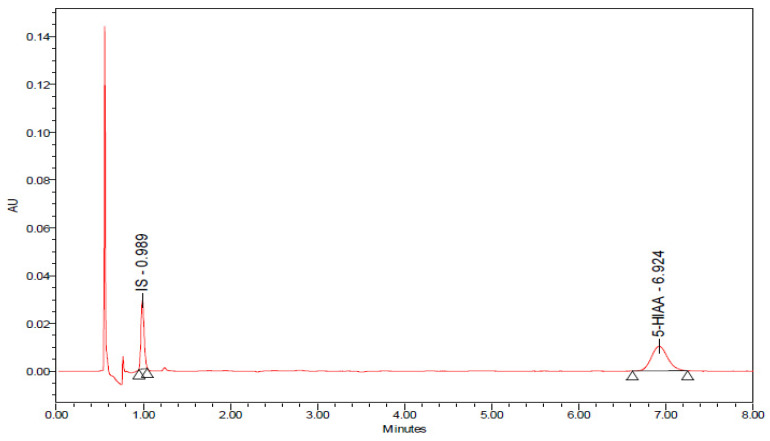
Chromatographs of a standard solution of 5-HT, 5-HIAA and QA. Abbreviations: 5-HT—5-hydroxytryptamine or serotonin, 5-HIAA—5-hydroxyindole acetic acid, QA—quinolinic acid, IS—internal standard and AU—arbitrary units.

We evaluated the effects of T1DM and chronic 8-OH-DPAT administration on the neurotransmitter, 5-HT, and its metabolite, 5-HIAA and QA. Figure 8 shows chromatographs of the standard solution, 5-HT and 5-HIAA, QA and internal standard, 3,4-dihroxybenzylamine. The retention time for 5-HT was 3.384 min, 5-HIAA was 6.924 min, and QA was 0.915 min.

Relative to control non-diabetic rats, 5-HT concentration in T1DM animals was elevated by 29.8% in the LSSC (F: 12.3, *p* = 0.0012, Table 1). On the other hand, the inactive metabolite 5-HIAA concentration was reduced by 11.7% (F: 4.76, *p* = 0.0301) STZ-induced T1DM.

The functionality of the serotonergic system, assessed 5-HIAA/5-HT ratio (5-HT turnover rate), was significantly suppressed in STZ-induced T1DM. As shown in Table 1, the 5-HIAA/5-HT ratio was reduced by 32.3% in the LSSC tissue isolated from T1DM animals relative to that of the control animals. Meanwhile, T1DM rats treated with 8-OH-DPAT showed a significant increase in the ratio (5-HIAA/5-HT) (F: 18.4, *p* = 0.0002).

Relative to control non-diabetic rats, the QA concentration in T1DM animals was elevated in the LSSC (Table 2). The concentration of QA was increased in T1DM rats when compared to their corresponding controls (Table 2), and treatment with 8-OH-DPAT reduced the concentration of QA by 76.3% in comparison to vehicle-treated T1DM rats (F: 66.1, *p* < 0.0001; Table 2).

A summary of all the results has been provided in Table 3.

## 3. Discussion

The results of this study demonstrate that chronic administration of 8-OH-DPAT (0.5 mg/kg, i.p.), a selective 5-HT1AR agonist, has antinociceptive (anti-hyperalgesic and anti-allodynic) activities in a T1DM-neuropathic pain rat model that were antagonized by WAY100635, a 5-HT1AR antagonist. In diabetic rats, 5-HT1AR transcript and protein levels were elevated in the RVM, LSSC, and DRG. Furthermore, colocalization of 5-HT1AR with SNAP-25 and PSD-95 indicated the presence of 5-HT1AR in pre- and postsynaptic LSSC tissue fractions. Chronic administration of 8-OH-DPAT downregulated the expression of 5-HT1AR in spinal presynaptic but not postsynaptic fractions. The gene expression levels of the methoxyindole pathway *Tph-2* and kynurenine pathway enzymes *Ido1*, *Ido2* and *Tdo* were significantly upregulated in the diabetic rats in comparison to those of control rats. Although treatment with 8-OH-DPAT did not alter the gene expression of *Tph-2*, it downregulated the expression of *Ido1*, *Ido2* and *Tdo* STZ-induced T1DM. Furthermore, the spinal release of 5-HT represented by the 5-HIAA/5-HT ratio was decreased, while QA concentration increased in the spinal cord as a function of T1DM. Treatment with 8-OH-DPAT increased the spinal release of 5-HT (**↑** 5-HIAA/5-HT) but decreased the concentration of QA STZ-induced T1DM.

Dysregulation of the serotoninergic system appears to be associated with several physiological and pathological states, including aging [31], postmenopausal syndrome [32], Parkinson’s disease [33], Alzheimer’s disease [34], depression [35], and more recently diabetes mellitus [36]. In this context, our study showed that the ratio of 5-HIAA/5-HT, an indicator of 5-HT release, was significantly decreased in STZ-treated diabetic rats. These changes are not unique to the diabetic state since similar results have been reported in aging [37,38,39,40,41,42,43,44,45] and in male and female rats with low levels of testosterone and estrogen, respectively [46,47,48,49,50]. This indicates that the aforementioned abnormalities appear not to be gender dependent.

We found that i.p. administration of 8-OH-DPAT elevated the nociceptive (thermal heat/cold and mechanical) threshold in STZ-induced T1DM rats. Consistent with our findings, some preclinical studies have shown that 8-OH-DPAT administration in the dorsal horn of the spinal cord reduced the activity of afferent sensory neurons, resulting in decreased NMDA receptor-dependent glutamate response and transmission of nociceptive signals [51,52]. Another study demonstrated that subcutaneous, intrathecal or intracerebroventricular administration of 8-OH-DPAT reduced pain sensitivity in the hot-plate test but had no effect on tail-flick latencies when administered via the same routes, indicating the involvement of 5-HT1AR both spinally and supraspinally [53]. In T1DM-neuropathic pain, decreased serotonergic neuronal firing and reduced availability of 5-HT in the dorsal horn of the spinal cord may reduce the inhibition of transmission of nociceptive signals and provoke increased perception of pain [11,54]. Recently, treatment with 8-OH-DPAT reversed both mechanical hypersensitivity and depression-like behavior in chronic constriction injury of the sciatic nerve in rats [30]. Furthermore, 8-OH-DPAT demonstrated an analgesic effect in the formalin model of tonic nociceptive pain [26]. No study, to the best of our knowledge, has yet evaluated the effects of 8-OH-DPAT in T1DM-associated thermal hyperalgesia, cold allodynia, and mechanical allodynia. The marked effects of 8-OH-DPAT in STZ-induced T1DM rats indicate that the serotonergic system, especially 5-HT1AR, is involved in T1DM-neuropathic pain that was further supported by the antagonization of 8-OH-DPAT’s antinociceptive activities by WAY100635. In the present study, acute (one week after STZ injection, i.p., 0.5 mg/kg) and chronic administration of 8-OH-DPAT (eight weeks after STZ injection, i.p., 0.5 mg/kg) produced similar antinociceptive effects and chronic administration of 8-OH-DPAT downregulated the expression of 5-HT1AR. We have recently demonstrated that chronic treatment with the alpha-2A adrenoceptor (α2A-AR) agonist, guanfacine, downregulates the expression of α2A-AR in STZ-induced T1DM rats [27].

Presumably, the time frame for antinociceptive effects after acute administration was too early for significant changes in the expression of 5-HT1AR. The activation of 5-HT1AR autoreceptors reduces the release of 5-HT; in this context, acute and chronic activation of these autoreceptors may have a different antinociceptive effect [55,56,57]. Activation of these receptors acutely would decrease 5-HT release, while chronic activation of these receptors may result in receptor desensitization and an increase in 5-HT release. In our findings, we showed that acute administration of 8-OH-DPAT to naïve rats did not demonstrate antinociceptive activity, whereas acute treatment with 8-OH-DPAT in diabetic rats reported antinociceptive activity. A possible explanation may be associated with its role in modulating peripheral expression of transient receptor protein vanilloid 1 (TRPV1) and/or transient receptor potential cation channel, subfamily A, member 1 (TRPA1), hyperalgesic nociceptors. Furthermore, preliminary results generated from our laboratory demonstrate that acute administration of 8-OH-DPAT (0.5 mg/kg) after one week of single i.p. STZ injection reverses mRNA upregulation of TRPV1 and TRPA1 in the DRG in comparison to STZ-induced T1DM rats.

Furthermore, changes in the expression of 5-HT1AR mRNA or protein levels in STZ-induced T1DM indicate malfunction/aberrant signaling of the serotonergic descending pathway. Nevertheless, it is well known that diabetes, if left uncontrolled, causes neuronal degeneration and atrophy in rodent models [58,59,60]. In our findings, we reported similar neuronal changes in both diabetic LSSC and DRG tissues; on the other hand, treatment with 8-OH-DPAT chronically improved the tissue morphology of diabetic rats to levels comparable to that observed in corresponding control rats. In the present study, 5-HT turnover rates were decreased in STZ-induced T1DM, similar to what has been described previously [61,62]. Therefore, it is possible that T1DM affects the activity of 5-HT neurons, although the underlying cellular mechanism responsible for this development is not yet established. In contrast to present observations, 5-HT turnover rates have been reported to be increased in a diabetic mouse brain [63]. This discrepancy may be due to species differences, regional distribution, and duration of the diabetic condition. 

In the present study, the transcripts of key enzymes, *Ido1*, *Ido2* and *Tdo*, involved in the kynurenic acid pathway were upregulated in STZ-induced T1DM, and this was reversed by treatment with 8-OH-DPAT. It has been found that IDO is activated under stress conditions, which consequently promotes abnormal tryptophan metabolism [64]. Tryptophan, kynurenine, and 3-hydroxykynurenine cross the blood–brain barrier (BBB) and kynurenic acid and QA are produced in astrocytes and microglia, respectively [65,66]. Kynurenic acid exerted neuroprotective and anti-inflammatory effects and maintained synaptic plasticity [67], whilst QA had neurotoxic effects by inhibiting glutamate reuptake, enhancing reactive oxygen species, BBB destruction and homeostasis imbalance [68]. Enhanced levels of kynurenine and its metabolites may induce neuroinflammation [69]. Therefore, a plausible explanation may be that neuroinflammation may develop in STZ-induced T1DM and lead to dysregulation of the serotonergic neuronal circuits. Furthermore, treatment with 8-OH-DPAT downregulated the expression of the enzymes involved in the kynurenine pathway, thus reducing the production of QA, which in turn is neurotoxic. This was confirmed with the HPLC results, where treatment with 8-OH-DPAT lowered the concentration of QA in STZ-induced T1DM. Therefore, a potential explanation may be that the neuroinflammatory mediators are involved in neuropathic pain progression. Indeed, the preliminary data from our laboratory demonstrated that chronic administration of 8-OH-DPAT suppresses the increase in pro-inflammatory mediators, tumor necrosis factor-alpha, interleukin-6, inducible-nitric oxide synthase and interleukin-1-beta in STZ-induced T1DM. Furthermore, the preliminary data from our laboratory show that in STZ-induced T1DM, 8-OH-DPAT reverses the decrease in anti-inflammatory mediators, CD206, tumor growth factor-beta and interleukin-10, and antioxidant markers, nuclear factor erythroid 2-related factor 2, heme oxygenase-1 and superoxide dismutase-2. The 5-HT1A autoreceptors are present in the serotonergic neurons. They inhibit neuronal firing and the release of 5-HT via the negative feedback mechanism [70,71,72]. In this context, a study has demonstrated that chronic administration of selective serotonin reuptake inhibitors causes internalization of the 5-HT1A autoreceptors of the raphe neurons, thereby increasing 5-HT levels in the synaptic cleft that bind to postsynaptic 5-HT1AR and modulate pain transmission [73]. In the present study, increased expression of presynaptic 5-HT1AR and 5-HT1AR coupled-Gα_i_ signaling in diabetic rats may have inhibited the release of 5-HT. The repeated administration of 8-OH-DPAT may have downregulated 5-HT1AR-coupled-Gα_i_ signaling in diabetic rats. Thus, it may have desensitized the presynaptic 5-HT1AR and diminished the autoinhibitory negative feedback mechanism.

## 4. Materials and Methods

### 4.1. Animals

Male Wistar rats (two–three months old; 300–400 g) were used in this study and were supplied by the Animal Resources Center at the Health Sciences Center (HSC), Kuwait University. The rats were kept in temperature-controlled rooms (24 ± 1 °C) with continuous access to food and water [74]. Experiments on animals were approved by the Ethical Committee for the use of Laboratory Animals in Teaching and in Research, HSC, Kuwait University (Ref: 23/VDR/EC/, Date 27 June 2021) in accordance with the guidelines of Directive 2010/63/EU of the European Parliament and of the Council on the protection of animals. All animal experiments were performed during the same period of the day (8:00 AM to 16:00 PM) to avoid any circadian changes in pharmacological effects.

### 4.2. Diabetes Induction

Type 1 diabetes mellitus was induced as previously described [27]. Briefly, rats were treated with streptozotocin (STZ, 55 mg/kg, intraperitoneally) or its vehicle (sodium citrate buffer, 50 mM, pH 4.5), and blood glucose concentrations in the tail-vein blood samples were measured using ACCU-CHEK test strips (Roche Diagnostics, Basel, Switzerland) three days after STZ injection. Rats with blood glucose concentrations greater than 250 mg/dL were included in the study. For the acute study, 8-OH-DPAT (0.25, 0.5 and 1.0 mg/kg, i.p.) was administered one week after STZ injection, whereas, for the chronic study, 8-OH-DPAT (0.5 mg/kg, i.p.) was administered eight weeks after STZ injection.

### 4.3. Drugs Administration

The 5-HT1AR agonist, 8-hydroxy-2-(propylamine) tetralin hydrobromide (8-OH-DPAT, Tocris, Bristol, UK), was administered intraperitoneally at doses of 0.25, 0.5, or 1.0 mg/kg. 8-OH-DPAT (0.5 mg/kg) was chosen as previously described [30,75], and 0.25 and 1.0 mg/kg were added for the dose–response curve. Freshly prepared 8-OH-DPAT was dissolved in normal saline and immediately used for each treatment in a volume of 1 mL/kg.

Diabetic rats were allocated on a random basis into four groups: I: T1DM+vehicle; II: T1DM+8-OH-DPAT-0.25; III: T1DM+8-OH-DPAT-0.5, and IV: T1DM+8-OH-DPAT-1. Similarly, control rats were distributed into groups of four: I: NC+vehicle; II: NC+8-OH-DPAT-0.25; III: NC+8-OH-DPAT-0.5, and IV: NC+8-OH-DPAT-1. The nociceptive threshold was recorded at different time points, for example, 0, 15, 30, 60, 90 and 120 min after drug administration. Furthermore, to assess the effects of the 5-HT1AR antagonist, WAY100635 (1 mg/kg; Tocris, Bristol, UK), diabetic rats were assigned randomly into four groups I: T1DM+vehicle; II: T1DM+WAY100635-1, III: T1DM+8-OH-DPAT-0.5, and IV: T1DM+WAY100635-1+8-OH-DPAT-0.5. WAY100635 was administered 15 min before 8-OH-DPAT injection for the thermal (heat/cold) and mechanical behavioral tests.

8-OH-DPAT was injected every day for a period of 14 days (chronically) following nociceptive behavior development to evaluate the antinociceptive effects of 8-OH-DPAT. The rats were randomly allocated into four groups: I: NC; II: NC+8-OH-DPAT-0.5; III: T1DM; and IV: T1DM+8-OH-DPAT-0.5. The nociceptive behavior was measured weekly at weeks 8, 9, 10, 11 and 12. However, on weeks 11 and 12, the drug was not administered to the rats. 8-OH-DPAT (0.5 mg/kg, i.p.) was administered at the same time each day for a period of two weeks (weeks 9 and 10). However, nociceptive behavior in response to thermal (heat/cold) and mechanical stimuli was recorded on a weekly basis.

### 4.4. Nociceptive Testing

The nociceptive behavior (thermal hyperalgesia and cold/mechanical allodynia) was evaluated using the Hargreaves test, cold plate and dynamic plantar aesthesiometer [27,76,77]. Briefly, in the Hargreaves test, a portable infrared (IR) heat source (IR intensity: 50 units) was applied vertically to the hind paw plantar surface. The time it took for the rat to withdraw its paw from the heat source was recorded as previously described [76]. To measure the thermal (cold) nociceptive threshold, the rat was placed on the cold plate (4 ± 0.1 °C). When the animal displayed a nociceptive response, such as jumping or licking, the reaction time was recorded [27]. Mechanical allodynia was assessed as previously described [77]. In this test, a linearly increasing force of 2.5 g/s, which is the mechanical stimulus, was applied to the hind paw using an aesthesiometer (Ugo Basil, Varese, Italy). The response to the withdrawal threshold was recorded at a cut-off force of 50 g or when the rat withdrew its paw.

### 4.5. Tissue Isolation

Rats were anesthetized using a mix of xylazine (Interchemie Werken, Holland, The Netherlands) and ketamine (Dutch Farm International, Holland, The Netherlands) and were sacrificed 12 weeks after STZ injection, as described previously [74]. The RVM tissue, located at the brain stem region, the LSSC (segment L1–L4) and DRG of the spinal nerves were dissected from the rats, immediately placed in liquid nitrogen to freeze the isolated tissues, and then powdered using a motor and pestle, and finally kept at −80 °C.

### 4.6. Analysis of Gene Expression by qRT-PCR

Expression of *Htr1a* mRNA and key enzymes of the serotonergic pathway were quantified relative to the expression of the housekeeping gene (*Actb*: β-actin) using real-time qRT-PCR. Total RNA was extracted from fresh powdered neuronal tissues using the acid guanidinium isothiocyanate–phenol–chloroform method [78], reverse-transcribed into cDNA and then real-time qRT-PCR was performed using QuantStudio™ 7 Flex Real-Time PCR System (Applied Biosystems, Waltham, MA, USA) as described previously [79]. The sequences of the primers used were: for *Actb* are forward, 5′-CCGCGAGTACAACCTTCTTG-3′ and reverse, 5′-GCAGCGATATCGTCAATCCAT-3′; for *Htr1a* are forward, 5′-CAGAGGAAGGTGCTCTTTGG-3′ and reverse, 5′-AAGAAGAGCCTGAACGGACA-3′, for *Tph-2* are forward, 5′-CTCCAAGCTTCGCATCACAG-3′ and reverse, 5′-AGCACTTCAGGAAGCGTACC-3′, for *Tdo* are forward, 5′-TGGGAACTAGATTCTGTTCG-3′ and reverse, 5′-TCGCTGCTGAAGTAAGAGCT-3′, for *Ido1* are forward, 5′-AGAAGTGGGCTTTGCTCTGC-3′ and reverse, 5′-TGGCAAGACCTTACGGACATCTC-3′, for *Ido2* are forward, 5′-AAGCTTATGGAGCCTCAAAGTCAGAGC-3′ and reverse, 5′-CTCGAGCTAAGCACCAGGACACAGG-3′. The comparative C_t_ method was used to calculate the relative gene expression to control [80]. Results were normalized to the housekeeping gene (*Actb*), and averages ± SEM are shown expressed relative to controls [81].

### 4.7. Western Blot Analysis and Immunohistochemistry- and Immunofluorescence-Staining of Tissue Sections

Analysis of Western blot was achieved as described previously [82]. Total protein concentration was determined using the Pierce BCA Protein Assay Kit (Thermo Fisher Scientific, Waltham, MA, USA) and 37.5 µg of protein was loaded onto 12 and 8% polyacrylamide gels. The polyvinylidene fluoride membranes (EMD Millipore Corporation, Billerica, MA, USA) were used to transfer proteins from the gel onto the membrane and immunoblotted with the corresponding primary and then with horseradish peroxidase-linked secondary antibodies (Table 4). The protein bands were developed using the Amersham ECL Prime Western Blotting Detection Reagent (GE Healthcare Life Sciences, Chicago, IL, USA) as previously described [83].

For immunohistochemistry experiments, immunohistochemistry was performed as described previously [84,85]. Briefly, fixed tissue sections were treated with primary antibody overnight, followed by secondary antibody horseradish peroxidase anti-rabbit IgG incubation at room temperature for one hour. Finally, the tissue sections were treated with 3,3′-diaminobenzidine for chromogen reactions. On the other hand, for immunofluorescence staining, the tissue sections were incubated with the appropriate primary antibodies overnight and then with fluorescence-labeled secondary antibodies (Table 4). Pictures were captured and analyzed using the Zeiss Axiovert 200 fluorescence microscope equipped with a CCD camera (Carl Zeiss Microscopy GmbH, Jena, Germany) and an IBAS 2000 image analyzer [83].

### 4.8. Extraction of Synaptosome and Pre-Synaptic and Post-Synaptic Fractions Isolation from Spinal Lumbar Region

A discontinuous sucrose gradient method was used to extract the synaptosomes from the spinal lumbar region as previously described [86]. Briefly, pre- and postsynaptic fractions were obtained following a sequence of differential centrifugation. The amount of protein was determined in each fraction and characterized using Western blot using appropriate primary and secondary antibodies (Table 4) as described in Section 4.7.

### 4.9. Levels of Spinal 5-HT and Its Metabolite, 5-HIAA, and QA Acid Using High-Performance Liquid Chromatography (HPLC) with Photodiode Array Detection (PDA)

The method described by Gu et al. (2016) [87] was used to detect the levels of 5-HT, 5-HIAA and QA in the LSSC of control, diabetic, and diabetic + 8-OH-DPAT-treated rats. Briefly, on the day of the experiment, stock solutions (10 µg/mL) of 5-HT, 5-HIAA and QA were prepared in 0.2 M perchloric acid solution and stored on ice in the dark. The neuronal tissues dissected from rats were weighed and homogenized in ice-cold 0.2 M perchloric acid solution (10 µL/mg tissue). After centrifugation at 12,000× *g* at 4 °C for 20 min, supernatants were collected for chromatographic separation. HPLC Equipment Waters 2535 Quaternary Gradient Module with a 15 uL fixed injection loop was used. The PDA detector used was a Waters 2998 Photodiode array controlled by Empower Software 3 for data acquisition and analysis. Chromatographic separation was performed using XBridge C18 5 um, 4.6 × 100 mm column (Waters XBridge, Wexford, Ireland). An amount of 5 mM perchloric acid solution containing 5% acetonitrile was used as a mobile phase, and the separation temperature was set at 12 °C with a flow rate adjusted to 2 mL/min [87].

### 4.10. Statistical Analyses

The D’Agostino–Pearson normality test was used to test for data normality, and parametric tests were used if the data passed the normality test; however, non-parametric tests were used if they failed the normality test. Statistical analyses were performed using unpaired Student’s *t*-test, Mann–Whitney test, one-way analysis of variance (ANOVA) followed by Dunnett’s multiple comparison post-tests, Kruskal–Wallis test followed by Dunn’s multiple comparisons test, or two-way repeated-measures ANOVA followed by Bonferroni’s multiple comparison post-tests using GraphPad Prism software (version 7.0) [88]. There were significant differences at *p* ≤ 0.05. The results obtained are expressed as the means ± SEM.

## 5. Conclusions

**Figure 9:**
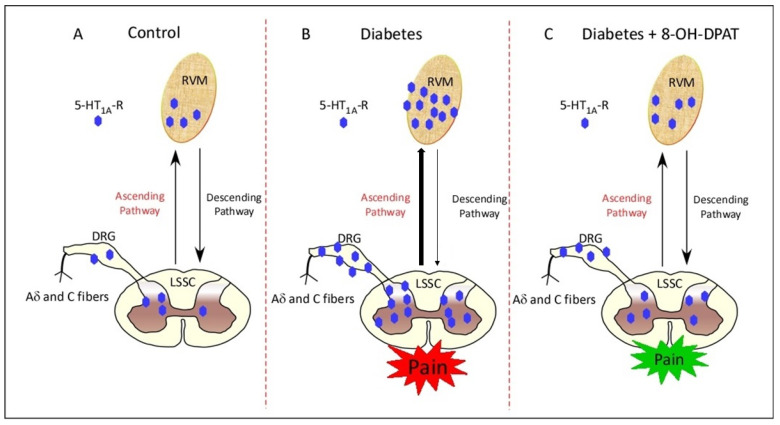
Schematic representation of presynaptic 5-HT1AR in STZ-induced T1DM, neuropathic pain and 8-OH-DPAT administration. When compared to the control status (**A**), diabetes enhances neuronal presynaptic expression of 5-HT1AR in the RVM, LSSC and DRG (**B**). 8-OH-DPAT administered chronically for 14 days reversed increased neuronal expression of presynaptic 5-HT1AR in this disease state (**C**). The upward arrow indicates the ascending pain transmission pathway, while the downward arrow indicates the descending pain transmission pathway. Pain in the red bubble indicates pain aggravation, and pain in the green bubble indicates pain alleviation. The thick and thin arrows in (**B**) represent hyperactive ascending pathways and hypoactive descending pathways. Abbreviations: 8-OH-DPAT—8-hydroxy-2-(dipropylamino)tetralin, 5-HT1AR—5-HT1A receptors, RVM—rostral ventromedial medulla, LSSC—lumbar segment of the spinal cord and DRG—dorsal root ganglia.

Overall, the present findings demonstrate alterations in the 5-HT1AR expression, and chronic administration of 8-OH-DPAT ameliorated this abnormality in STZ-induced T1DM. Moreover, treatment with 8-OH-DPAT alleviated T1DM-associated nociceptive behavior. These effects of 8-OH-DPAT at both the behavioral and biochemical levels elucidated in the context of T1DM were presented schematically in Figure 9. Furthermore, chronic administration of 8-OH-DPAT may have downregulated 5-HT1AR-coupled-Gα_i_ signaling in diabetic rats and diminished the autoinhibitory negative feedback mechanism. Overall, taking into consideration the present data, future studies are warranted to examine whether 8-OH-DPAT is able to ameliorate DNP clinically.

## 6. Limitations of the Study

The H&E and immunohistochemical staining data outlined in our manuscript are simply qualitative in nature and no solid conclusive information can be drawn out of them.

## Figures and Tables

**Table 1:** 8-hydroxy-2-(dipropylamino)tetralin dependent changes in LSSC 5-HT metabolism STZ-induced T1DM.

Treatment	5-HIAA (ng/mL)	5-HT (ng/mL)	5-HIAA/5-HT (ng/mL)
NC	12.0 ± 0.302	14.1 ± 0.364	0.854 ± 0.03
T1DM	10.6 ± 0.484 *	18.3 ± 0.510 *	0.578 ± 0.03 *
T1DM + 8-OH-DPAT	11.5 ± 0.06	16.7 ± 0.843	0.689 ± 0.03 **

Each bar represents the mean ± SEM of values obtained from five animals/group. * Diabetic animals were significantly different from corresponding control values at *p* ≤ 0.05. ** 8-OH-DPAT-treated diabetic animals were significantly different from corresponding diabetic animals at *p* ≤ 0.05. One-way ANOVA followed by Dunnett’s post-test. Abbreviations: 5-HT—5-hydroxytryptamine or serotonin, 5-HIAA—5-hydroxyindole acetic acid, NC—normal control, T1DM—type 1 diabetes mellitus, LSSC—lumbar segment of the spinal cord and 8-OH-DPAT—8-hydroxy-2-(dipropylamino)tetralin.

**Table 2:** 8-hydroxy-2-(dipropylamino)tetralin dependent changes in LSSC QA concentration STZ-induced T1DM.

Treatment	QA (ng/mL)
NC	147.4 ± 7.52
T1DM	373.9 ± 30.2 *
T1DM + 8-OH-DPAT	88.5 ± 7.85 **

Each bar represents the mean ± SEM of values obtained from five animals/group. * Diabetic animals were significantly different from corresponding control values at *p* ≤ 0.05. ** 8-OH-DPAT-treated diabetic animals were significantly different from corresponding diabetic animals at *p* ≤ 0.05. One-way ANOVA followed by Dunnett’s post-test. Abbreviations: 5-HT—5-hydroxytryptamine or serotonin, 5-HIAA—5-hydroxyindole acetic acid, NC—normal control, T1DM—type 1 diabetes mellitus, LSSC—lumbar segment of the spinal cord, QA—quinolinic acid and 8-OH-DPAT—8-hydroxy-2-(dipropylamino)tetralin.

**Table 3:** Summary of results.

T1DM	Effects of Chronic 8-OH-DPAT (0.5 mg/kg, i.p.) Administration
↓ Pain threshold	↑ Pain threshold
Neuronal degeneration (LSSC and DRG)	Improved neuronal degeneration (LSSC and DRG)
↓ 5-HT1AR mRNA and protein expression (RVM, LSSC and DRG)	↑ 5-HT1AR mRNA and protein expression (RVM, LSSC and DRG)
↑ Presynaptic 5-HT1AR	↓ Presynaptic 5-HT1AR
↑ Gαi	↓ Gαi
↑ Tph-2 mRNA (RVM, LSSC and DRG)	No change in Tph-2 mRNA (RVM, LSSC and DRG)
↑ Tdo/Ido-1/2 mRNA (LSSC)	↓ Tdo/Ido-1/2 mRNA (LSSC)
↓ 5-HIAA/5-HT (LSSC)	↑ 5-HIAA/5-HT (LSSC)
↑ QA (LSSC)	↓ QA (LSSC)

↑ means increased, while ↓ means decreased.

**Table 4:** Antibodies used in the study.

Antibody (Vendor)	Dilution
5-HT1A receptor (5-HT1AR) (Abcam, Cambridge, MA, USA)	1:500, 1:100
SNAP-25 (Santa Cruz Biotechnology, Inc., Dallas, TX, USA)	1:1000, 1:100
PSD-95 (Novus Biologicals, Centennial CO, USA)	1:2000, 1:100
Secondary antibody, horseradish peroxidase (HRP) anti-rabbit IgG (Cell Signaling Technology, Danvers, MA, USA)	1:3000
Secondary antibody, HRP-linked anti-mouse IgG (Cell Signaling Technology, Inc., Danvers, MA, USA)	1:3000
AlexaFluor-488-labeled goat anti-mouse IgG antibody (Invitrogen, Carlsbad, CA, USA)	1:100
AlexaFluor-546 labeled goat anti-rabbit IgG antibody (Invitrogen, Carlsbad, CA, USA)	1:100

## Data Availability

Data is contained within the article.

## References

[1] Hicks C.W., Selvin E. (2019). Epidemiology of Peripheral Neuropathy and Lower Extremity Disease in Diabetes. Curr. Diabetes Rep..

[2] van Hecke O., Austin S.K., Khan R.A., Smith B.H., Torrance N. (2014). Neuropathic pain in the general population: A systematic review of epidemiological studies. Pain.

[3] Javed S., Alam U., Malik R.A. (2015). Treating Diabetic Neuropathy: Present Strategies and Emerging Solutions. Rev. Diabet. Stud..

[4] Jordan L.M., Kenshalo D.R., Martin F.R., Haber L.H., Willis W.D. (1978). Depression of primate spinothalamic tract neurons by iontophoretic application of 5-hydroxytryptamine. Pain.

[5] Bardin L. (2011). The complex role of serotonin and 5-HT receptors in chronic pain. Behav. Pharmacol..

[6] Martin S.L., Power A., Boyle Y., Anderson I.M., Silverdale M.A., Jones A.K.P. (2017). 5-HT modulation of pain perception in humans. Psychopharmacology.

[7] Millan M.J. (2002). Descending control of pain. Prog. Neurobiol..

[8] Dogrul A., Ossipov M.H., Porreca F. (2009). Differential mediation of descending pain facilitation and inhibition by spinal 5HT-3 and 5HT-7 receptors. Brain Res..

[9] Green M.G., Scarth J., Dickenson A. (2000). An excitatory role for 5-HT in spinal inflammatory nociceptive transmission; state-dependent actions via dorsal horn 5-HT(3) receptors in the anaesthetized rat. Pain.

[10] Rahman W., Suzuki R., Rygh L.J., Dickenson A.H. (2004). Descending serotonergic facilitation mediated through rat spinal 5HT3 receptors is unaltered following carrageenan inflammation. Neurosci. Lett..

[11] Haleem D.J. (2019). Targeting Serotonin1A Receptors for Treating Chronic Pain and Depression. Curr. Neuropharmacol..

[12] Jacobs B.L., Azmitia E.C. (1992). Structure and function of the brain serotonin system. Physiol. Rev..

[13] Doherty M.D., Pickel V.M. (2001). Targeting of serotonin 1A receptors to dopaminergic neurons within the parabrachial subdivision of the ventral tegmental area in rat brain. J. Comp. Neurol..

[14] Pompeiano M., Palacios J.M., Mengod G. (1992). Distribution and cellular localization of mRNA coding for 5-HT1A receptor in the rat brain: Correlation with receptor binding. J. Neurosci..

[15] Perrin F.E., Gerber Y.N., Teigell M., Lonjon N., Boniface G., Bauchet L., Rodriguez J.J., Hugnot J.P., Privat A.M. (2011). Anatomical study of serotonergic innervation and 5-HT(1A) receptor in the human spinal cord. Cell Death Dis..

[16] Otoshi C.K., Walwyn W.M., Tillakaratne N.J., Zhong H., Roy R.R., Edgerton V.R. (2009). Distribution and localization of 5-HT(1A) receptors in the rat lumbar spinal cord after transection and deafferentation. J. Neurotrauma.

[17] Braz J.M., Basbaum A.I. (2008). Genetically expressed transneuronal tracer reveals direct and indirect serotonergic descending control circuits. J. Comp. Neurol..

[18] Seyrek M., Kahraman S., Deveci M.S., Yesilyurt O., Dogrul A. (2010). Systemic cannabinoids produce CB(1)-mediated antinociception by activation of descending serotonergic pathways that act upon spinal 5-HT(7) and 5-HT(2A) receptors. Eur. J. Pharmacol..

[19] Gal E.M., Sherman A.D. (1980). L-kynurenine: Its synthesis and possible regulatory function in brain. Neurochem. Res..

[20] Rubi B., Maechler P. (2010). Minireview: New roles for peripheral dopamine on metabolic control and tumor growth: Let’s seek the balance. Endocrinology.

[21] Malek Z.S., Dardente H., Pevet P., Raison S. (2005). Tissue-specific expression of tryptophan hydroxylase mRNAs in the rat midbrain: Anatomical evidence and daily profiles. Eur. J. Neurosci..

[22] Yabut J.M., Crane J.D., Green A.E., Keating D.J., Khan W.I., Steinberg G.R. (2019). Emerging Roles for Serotonin in Regulating Metabolism: New Implications for an Ancient Molecule. Endocr. Rev..

[23] Oxenkrug G.F. (2007). Genetic and hormonal regulation of tryptophan kynurenine metabolism: Implications for vascular cognitive impairment, major depressive disorder, and aging. Ann. N. Y. Acad. Sci..

[24] Guidetti P., Amori L., Sapko M.T., Okuno E., Schwarcz R. (2007). Mitochondrial aspartate aminotransferase: A third kynurenate-producing enzyme in the mammalian brain. J. Neurochem..

[25] Guidetti P., Hoffman G.E., Melendez-Ferro M., Albuquerque E.X., Schwarcz R. (2007). Astrocytic localization of kynurenine aminotransferase II in the rat brain visualized by immunocytochemistry. Glia.

[26] Bardin L., Tarayre J.P., Koek W., Colpaert F.C. (2001). In the formalin model of tonic nociceptive pain, 8-OH-DPAT produces 5-HT1A receptor-mediated, behaviorally specific analgesia. Eur. J. Pharmacol..

[27] Munawar N., Nader J., Khadadah N.H., Al Madhoun A., Al-Ali W., Varghese L.A., Masocha W., Al-Mulla F., Bitar M.S. (2022). Guanfacine Normalizes the Overexpression of Presynaptic alpha-2A Adrenoceptor Signaling and Ameliorates Neuropathic Pain in a Chronic Animal Model of Type 1 Diabetes. Pharmaceutics.

[28] Pytliak M., Vargova V., Mechirova V., Felsoci M. (2011). Serotonin receptors—From molecular biology to clinical applications. Physiol. Res..

[29] Demeulemeester H., Feys H., Goris I., Zwaenepoel I., de Weerdt W., de Sutter P., Gybels J., Plets C., Nuttin B. (2001). Effect of the serotonin agonist 8-OH-DPAT on the sensorimotor system of the rat. Pharmacol. Biochem. Behav..

[30] Hu B., Doods H., Treede R.D., Ceci A. (2016). Duloxetine and 8-OH-DPAT, but not fluoxetine, reduce depression-like behaviour in an animal model of chronic neuropathic pain. Neurosci. Lett..

[31] Rodriguez J.J., Noristani H.N., Verkhratsky A. (2012). The serotonergic system in ageing and Alzheimer’s disease. Prog. Neurobiol..

[32] Schneider-Matyka D., Jurczak A., Szkup M., Samochowiec A., Grzywacz A., Wieder-Huszla S., Grochans E. (2017). The influence of the serotonergic system on the personality and quality of life of postmenopausal women. Clin. Interv. Aging.

[33] Politis M., Niccolini F. (2015). Serotonin in Parkinson’s disease. Behav. Brain Res..

[34] Lalut J., Karila D., Dallemagne P., Rochais C. (2017). Modulating 5-HT(4) and 5-HT(6) receptors in Alzheimer’s disease treatment. Future Med. Chem..

[35] Yohn C.N., Gergues M.M., Samuels B.A. (2017). The role of 5-HT receptors in depression. Mol. Brain.

[36] Cai X., Gu Z., Zhong P., Ren Y., Yan Z. (2002). Serotonin 5-HT1A receptors regulate AMPA receptor channels through inhibiting Ca2+/calmodulin-dependent kinase II in prefrontal cortical pyramidal neurons. J. Biol. Chem..

[37] Palmer A.M., DeKosky S.T. (1993). Monoamine neurons in aging and Alzheimer’s disease. J. Neural Transm. Gen. Sect..

[38] Dillon K.A., Gross-Isseroff R., Israeli M., Biegon A. (1991). Autoradiographic analysis of serotonin 5-HT1A receptor binding in the human brain postmortem: Effects of age and alcohol. Brain Res..

[39] Marcusson J., Oreland L., Winblad B. (1984). Effect of age on human brain serotonin (S-1) binding sites. J. Neurochem..

[40] Tauscher J., Verhoeff N.P., Christensen B.K., Hussey D., Meyer J.H., Kecojevic A., Javanmard M., Kasper S., Kapur S. (2001). Serotonin 5-HT1A receptor binding potential declines with age as measured by [11C]WAY-100635 and PET. Neuropsychopharmacology.

[41] Steinbusch H.W., Van Luijtelaar M.G., Dijkstra H., Nijssen A., Tonnaer J.A. (1990). Aging and regenerative capacity of the rat serotonergic system. A morphological, neurochemical and behavioral analysis after transplantation of fetal raphe cells. Ann. N. Y. Acad. Sci..

[42] Venero J.L., de la Roza C., Machado A., Cano J. (1993). Age-related changes on monoamine turnover in hippocampus of rats. Brain Res..

[43] Birthelmer A., Lazaris A., Schweizer T., Jackisch R., Cassel J.C. (2003). Presynaptic regulation of neurotransmitter release in the cortex of aged rats with differential memory impairments. Pharmacol. Biochem. Behav..

[44] Birthelmer A., Stemmelin J., Jackisch R., Cassel J.C. (2003). Presynaptic modulation of acetylcholine, noradrenaline, and serotonin release in the hippocampus of aged rats with various levels of memory impairments. Brain Res. Bull..

[45] Gur R.E., Gur R.C. (2002). Gender differences in aging: Cognition, emotions, and neuroimaging studies. Dialogues Clin. Neurosci..

[46] Pandaranandaka J., Poonyachoti S., Kalandakanond-Thongsong S. (2009). Differential effects of exogenous and endogenous estrogen on anxiety as measured by elevated T-maze in relation to the serotonergic system. Behav. Brain Res..

[47] Thiblin I., Finn A., Ross S.B., Stenfors C. (1999). Increased dopaminergic and 5-hydroxytryptaminergic activities in male rat brain following long-term treatment with anabolic androgenic steroids. Br. J. Pharmacol..

[48] Harman S.M., Metter E.J., Tobin J.D., Pearson J., Blackman M.R. (2001). Longitudinal effects of aging on serum total and free testosterone levels in healthy men. Baltimore Longitudinal Study of Aging. J. Clin. Endocrinol. Metab..

[49] Mohr B.A., Guay A.T., O’Donnell A.B., McKinlay J.B. (2005). Normal, bound and nonbound testosterone levels in normally ageing men: Results from the Massachusetts Male Ageing Study. Clin. Endocrinol..

[50] Horstman A.M., Dillon E.L., Urban R.J., Sheffield-Moore M. (2012). The role of androgens and estrogens on healthy aging and longevity. J. Gerontol. Biol. Sci. Med. Sci..

[51] Gjerstad J., Tjolsen A., Hole K. (1996). The effect of 5-HT1A receptor stimulation on nociceptive dorsal horn neurones in rats. Eur. J. Pharmacol..

[52] Mjellem-Joly N., Lund A., Berge O.G., Hole K. (1992). Intrathecal co-administration of substance P and NMDA augments nociceptive responses in the formalin test. Pain.

[53] Fasmer O.B., Berge O.G., Post C., Hole K. (1986). Effects of the putative 5-HT1A receptor agonist 8-OH-2-(di-n-propylamino)tetralin on nociceptive sensitivity in mice. Pharmacol. Biochem. Behav..

[54] Haleem D.J., Nawaz S. (2017). Inhibition of Reinforcing, Hyperalgesic, and Motor Effects of Morphine by Buspirone in Rats. J. Pain..

[55] Barnes N.M., Sharp T. (1999). A review of central 5-HT receptors and their function. Neuropharmacology.

[56] Uys M.M., Shahid M., Harvey B.H. (2017). Therapeutic Potential of Selectively Targeting the alpha(2C)-Adrenoceptor in Cognition, Depression, and Schizophrenia-New Developments and Future Perspective. Front. Psychiatry.

[57] Albert P.R., Vahid-Ansari F. (2019). The 5-HT1A receptor: Signaling to behavior. Biochimie.

[58] Cheng C., Zochodne D.W. (2003). Sensory neurons with activated caspase-3 survive long-term experimental diabetes. Diabetes.

[59] Kishi M., Tanabe J., Schmelzer J.D., Low P.A. (2002). Morphometry of dorsal root ganglion in chronic experimental diabetic neuropathy. Diabetes.

[60] Kobayashi M., Chandrasekhar A., Cheng C., Martinez J.A., Ng H., de la Hoz C., Zochodne D.W. (2017). Diabetic polyneuropathy, sensory neurons, nuclear structure and spliceosome alterations: A role for CWC22. Dis. Model. Mech..

[61] Bellush L.L., Reid S.G., North D. (1991). The functional significance of biochemical alterations in streptozotocin-induced diabetes. Physiol. Behav..

[62] Bitar M., Koulu M., Rapoport S.I., Linnoila M. (1986). Diabetes-induced alteration in brain monoamine metabolism in rats. J. Pharmacol. Exp. Ther..

[63] Miyata S., Hirano S., Kamei J. (2004). Diabetes attenuates the antidepressant-like effect mediated by the activation of 5-HT1A receptor in the mouse tail suspension test. Neuropsychopharmacology.

[64] Heisler J.M., O’Connor J.C. (2015). Indoleamine 2,3-dioxygenase-dependent neurotoxic kynurenine metabolism mediates inflammation-induced deficit in recognition memory. Brain Behav. Immun..

[65] Owe-Young R., Webster N.L., Mukhtar M., Pomerantz R.J., Smythe G., Walker D., Armati P.J., Crowe S.M., Brew B.J. (2008). Kynurenine pathway metabolism in human blood-brain-barrier cells: Implications for immune tolerance and neurotoxicity. J. Neurochem..

[66] Platten M., Nollen E.A.A., Rohrig U.F., Fallarino F., Opitz C.A. (2019). Tryptophan metabolism as a common therapeutic target in cancer, neurodegeneration and beyond. Nat. Rev. Drug Discov..

[67] Potter M.C., Elmer G.I., Bergeron R., Albuquerque E.X., Guidetti P., Wu H.Q., Schwarcz R. (2010). Reduction of endogenous kynurenic acid formation enhances extracellular glutamate, hippocampal plasticity, and cognitive behavior. Neuropsychopharmacology.

[68] Ferreira F.S., Schmitz F., Marques E.P., Siebert C., Wyse A.T.S. (2020). Intrastriatal Quinolinic Acid Administration Impairs Redox Homeostasis and Induces Inflammatory Changes: Prevention by Kynurenic Acid. Neurotox. Res..

[69] Heyes M.P., Saito K., Crowley J.S., Davis L.E., Demitrack M.A., Der M., Dilling L.A., Elia J., Kruesi M.J., Lackner A. (1992). Quinolinic acid and kynurenine pathway metabolism in inflammatory and non-inflammatory neurological disease. Brain.

[70] Adayev T., Ranasinghe B., Banerjee P. (2005). Transmembrane signaling in the brain by serotonin, a key regulator of physiology and emotion. Biosci. Rep..

[71] Hjorth S., Bengtsson H.J., Kullberg A., Carlzon D., Peilot H., Auerbach S.B. (2000). Serotonin autoreceptor function and antidepressant drug action. J. Psychopharmacol..

[72] Polter A.M., Li X. (2010). 5-HT1A receptor-regulated signal transduction pathways in brain. Cell Signal.

[73] Riad M., Zimmer L., Rbah L., Watkins K.C., Hamon M., Descarries L. (2004). Acute treatment with the antidepressant fluoxetine internalizes 5-HT1A autoreceptors and reduces the in vivo binding of the PET radioligand [18F]MPPF in the nucleus raphe dorsalis of rat. J. Neurosci..

[74] Bitar M.S., Bajic K.T., Farook T., Thomas M.I., Pilcher C.W. (1999). Spinal cord noradrenergic dynamics in diabetic and hypercortisolaemic states. Brain Res..

[75] Prow M.R., Martin K.F., Heal D.J. (1996). 8-OH-DPAT-induced mydriasis in mice: A pharmacological characterisation. Eur. J. Pharmacol..

[76] Hargreaves K., Dubner R., Brown F., Flores C., Joris J. (1988). A new and sensitive method for measuring thermal nociception in cutaneous hyperalgesia. Pain.

[77] Thangamani D., Edafiogho I.O., Masocha W. (2013). The anticonvulsant enaminone E139 attenuates paclitaxel-induced neuropathic pain in rodents. Sci. World J..

[78] Hatzis P., Al-Madhoon A.S., Jullig M., Petrakis T.G., Eriksson S., Talianidis I. (1998). The intracellular localization of deoxycytidine kinase. J. Biol. Chem..

[79] Masocha W. (2009). Systemic lipopolysaccharide (LPS)-induced microglial activation results in different temporal reduction of CD200 and CD200 receptor gene expression in the brain. J. Neuroimmunol..

[80] Abdel-Halim S.M., Al Madhoun A., Nizam R., Melhem M., Cherian P., Al-Khairi I., Haddad D., Abu-Farha M., Abubaker J., Bitar M.S. (2020). Increased Plasma Levels of Adenylate Cyclase 8 and cAMP Are Associated with Obesity and Type 2 Diabetes: Results from a Cross-Sectional Study. Biology.

[81] Al-Roub A., Al Madhoun A., Akhter N., Thomas R., Miranda L., Jacob T., Al-Ozairi E., Al-Mulla F., Sindhu S., Ahmad R. (2021). IL-1beta and TNFalpha Cooperativity in Regulating IL-6 Expression in Adipocytes Depends on CREB Binding and H3K14 Acetylation. Cells.

[82] Gil-Recio C., Montori S., Al Demour S., Ababneh M.A., Ferres-Padro E., Marti C., Ferres-Amat E., Barajas M., Al Madhoun A., Atari M. (2021). Chemically Defined Conditions Mediate an Efficient Induction of Dental Pulp Pluripotent-Like Stem Cells into Hepatocyte-Like Cells. Stem Cells Int..

[83] Al Madhoun A., Haddad D., Al Tarrah M., Jacob S., Al-Ali W., Nizam R., Miranda L., Al-Rashed F., Sindhu S., Ahmad R. (2021). Microarray analysis reveals ONC201 mediated differential mechanisms of CHOP gene regulation in metastatic and nonmetastatic colorectal cancer cells. Sci. Rep..

[84] Akhter N., Wilson A., Thomas R., Al-Rashed F., Kochumon S., Al-Roub A., Arefanian H., Al-Madhoun A., Al-Mulla F., Ahmad R. (2021). ROS/TNF-alpha Crosstalk Triggers the Expression of IL-8 and MCP-1 in Human Monocytic THP-1 Cells via the NF-kappaB and ERK1/2 Mediated Signaling. Int. J. Mol. Sci..

[85] Kochumon S., Al Madhoun A., Al-Rashed F., Thomas R., Sindhu S., Al-Ozairi E., Al-Mulla F., Ahmad R. (2020). Elevated adipose tissue associated IL-2 expression in obesity correlates with metabolic inflammation and insulin resistance. Sci. Rep..

[86] Lopes J.P., Morato X., Souza C., Pinhal C., Machado N.J., Canas P.M., Silva H.B., Stagljar I., Gandia J., Fernandez-Duenas V. (2015). The role of parkinson’s disease-associated receptor GPR37 in the hippocampus: Functional interplay with the adenosinergic system. J. Neurochem..

[87] Gu M.J., Jeon J.H., Oh M.S., Hong S.P. (2016). Measuring levels of biogenic amines and their metabolites in rat brain tissue using high-performance liquid chromatography with photodiode array detection. Arch. Pharm. Res..

[88] Al Madhoun A.S., Mehta V., Li G., Figeys D., Wiper-Bergeron N., Skerjanc I.S. (2011). Skeletal myosin light chain kinase regulates skeletal myogenesis by phosphorylation of MEF2C. EMBO J..

